# The Gut–Brain Axis in Brain Tumors: Insights into Tumor Development, Progression, and Therapy

**DOI:** 10.3390/biomedicines13092172

**Published:** 2025-09-05

**Authors:** Sarah Adriana Scuderi, Alessio Ardizzone, Elsa Calcaterra, Nicoletta Palermo, Fabiola De Luca, Antonio Catalfamo, Emanuela Esposito, Anna Paola Capra

**Affiliations:** 1 Department of Chemical, Biological, Pharmaceutical and Environmental Sciences, University of Messina, Viale Ferdinando Stagno D’Alcontres 31, 98166 Messina, ME, Italy; sascuderi@unime.it (S.A.S.); alessio.ardizzone@unime.it (A.A.); elsa.calcaterra@unime.it (E.C.); fabiola.deluca@unime.it (F.D.L.); ntncatalfamo@gmail.com (A.C.); annapaola.capra@unime.it (A.P.C.); 2 Department of Biomedical and Dental Sciences and Morphofunctional Imaging, University of Messina, Via Consolare Valeria 1, 98125 Messina, ME, Italy; nicoletta.palermo1@unime.it

**Keywords:** gut–brain axis, microbiota, brain tumors, glioblastoma, therapy

## Abstract

The gut–brain axis refers to the bidirectional communication network linking the gut microbiota and the central nervous system (CNS). Recent research has highlighted the critical role of gut microbiota in influencing brain health, neurogenesis, and neuroinflammation. In the context of brain tumors, especially gliomas, the gut–brain axis plays a significant role in tumor development, progression, and response to therapy. Gut dysbiosis, characterized by an imbalance in microbiota composition, has been linked to chronic inflammation, immune suppression, and altered blood–brain barrier (BBB) permeability, key factors in glioma pathogenesis. Gut-derived metabolites such as short-chain fatty acids (SCFAs) and neurotransmitters can either promote or inhibit tumor growth, impacting the tumor microenvironment (TME) and immune responses. Emerging evidence suggests that microbiome modulation, through strategies such as probiotics, prebiotics, and dietary interventions, may enhance anti-tumor immunity and improve the efficacy of conventional treatments like chemotherapy, radiotherapy, and immunotherapy. This review examines the interactions between gut microbiota and brain tumors, focusing on how microbiota alterations may influence tumor biology and therapeutic outcomes. Understanding the mechanisms of the gut–brain axis could lead to novel adjunctive therapies in neuro-oncology, offering new prospects for personalized treatment strategies in brain tumor management.

## 1. Introduction

The gut–brain axis is a bidirectional communication network linking the gastrointestinal (GI) system and the central nervous system (CNS) through neural, endocrine, and immune pathways [1]. This intricate system plays a key role in maintaining homeostasis and regulating physiological processes, including neurogenesis, immune responses, and inflammation. Recent research has uncovered the profound influence of gut microbiota, the diverse community of microorganisms residing in the GI tract, on brain health, and the pathogenesis of neurological diseases, including brain tumors [2,3]. Brain tumors, particularly gliomas, remain among the most challenging malignancies due to their aggressive nature and limited treatment options [4]. Emerging evidence suggests that gut microbiota composition and its metabolic byproducts can modulate the tumor microenvironment (TME), influence immune system function, and impact treatment efficacy [5,6]. Dysbiosis, or the imbalance of gut microbiota, has been associated with chronic inflammation, immunosuppression, and altered blood–brain barrier (BBB) permeability that can contribute to glioma initiation and progression [7]. Additionally, gut-derived metabolites, such as short-chain fatty acids (SCFAs) and neurotransmitters, may have direct and indirect effects on glioma biology, either promoting or inhibiting tumor growth [2,8]. Understanding the interplay between the gut microbiome and brain tumors presents novel opportunities for therapeutic interventions. Strategies such as probiotics, prebiotics, dietary modifications, and fecal microbiota transplantation (FMT) are being explored to restore microbial balance and enhance anti-tumor immunity [9]. Moreover, the gut microbiota’s role in shaping responses to conventional treatments, including chemotherapy, radiotherapy, and immunotherapy, underscores its potential as a modifiable factor in personalized cancer therapy [10]. This review aims to explore the influence of the gut–brain axis on brain tumors by examining the mechanisms through which gut microbiota regulates tumor development and progression. Additionally, we will discuss how gut microbiome modulation could serve as a promising avenue for improving therapeutic outcomes in brain tumor patients. By elucidating these complex interactions, we may unlock new insights into innovative treatment approaches that integrate microbiota-targeted strategies in neuro-oncology.

## 2. A Comprehensive Overview of Brain Tumors: From Astrocytomas to Meningiomas

Brain tumors constitute a highly heterogeneous group of neoplasms that differ widely in their cellular origin, biological behavior, and clinical outcomes [11,12,13]. Among them, gliomas constitute the most frequent type and were historically classified according to histopathological features. This morphology-based approach, however, was often limited by interobserver variability and insufficient prognostic accuracy. Traditionally, gliomas were graded along a spectrum ranging from low-grade pilocytic astrocytoma (Grade I) to the highly malignant glioblastoma (GBM, Grade IV) [14].

The landmark 2016 World Health Organization (WHO) classification introduced molecular markers, particularly IDH mutations and 1p/19q-codeletion status, establishing a more reliable and prognostically meaningful framework. Later, the 2021 revision further advanced this approach by fully integrating molecular diagnostics, including tools such as immunohistochemistry (IHC), next-generation sequencing (NGS), fluorescence in situ hybridization (FISH), PCR-based mutation analysis, and methylation profiling, thereby improving both the accuracy of glioma classification and its value for therapeutic decision-making [14,15].

From a biological perspective, glial tumors originate from cells of glial lineage and remain the most common primary brain tumors. They are traditionally categorized into low- and high-grade lesions based on their growth pattern and clinical behavior. Low-grade gliomas, including pilocytic astrocytoma (Grade I) and diffuse astrocytoma (Grade II), are generally slow-growing and associated with a more favorable prognosis. In contrast, high-grade gliomas, such as anaplastic astrocytoma (Grade III) and GBM (Grade IV), display rapid growth, aggressive biological behavior, and poorer prognosis [11,16]. Among them, GBM is the most aggressive and lethal primary brain tumor in adults [4,11] with an incidence of approximately 3.2 cases per 100,000 people annually [17,18]. Gliomas classification according to WHO and key diagnostic genes [14] is shown in Table 1.

In summary, the epidemiological landscape of brain tumors reveals a complex picture where the overall incidence and mortality are driven by the highly aggressive nature of astrocytoma, especially in the context of GBMs [19]. The distinct biological behaviors and varying degrees of aggressiveness among these tumor types underscore the critical need for continued research into tailored therapeutic strategies that address both the molecular heterogeneity and clinical challenges posed by these neoplasms.

### 2.1. Astrocytomas

Astrocytomas are primary brain tumors arising from astrocytes, a type of glial cell [11]. Astrocytes are involved in neurotransmitter regulation, ion homeostasis, and synaptic support [11]. In their neoplastic state, these cells lose regulatory control, which contributes to tumor growth and the eventual malignant transformation observed in higher-grade astrocytomas [20]. Astrocytomas exhibit a broad spectrum of behavior ranging from low-grade lesions to highly aggressive malignancies. In detail, astrocytomas are classified as follows: pilocytic astrocytoma and subependymal giant cell (Grade I), diffuse astrocytomas (Grade II), pleomorphic xanthastrocytomas (Grades II and III), anaplastic astrocytoma (Grade III), and glioblastoma (GBM) (Grade IV) [11]. These various degrees of histological variability correspond to different degrees of malignancy, which is given both by the speed of growth and the ability to reform themselves after surgical removal [11]. Therefore, the evaluation of the degree is an important parameter for both prognosis and therapy.

#### 2.1.1. Non-Glioblastoma Astrocytomas

Non-glioblastoma astrocytomas, which primarily encompass Grade II (diffuse astrocytomas) and Grade III (anaplastic astrocytomas), represent a heterogeneous group of primary brain tumors with a variable clinical course. Epidemiologically, these tumors have a lower incidence compared to GBMs [16]. Despite their lower initial aggressiveness, these tumors can progress over time, sometimes undergoing malignant transformation to higher grades, which contributes to significant long-term morbidity and mortality.

The integration of molecular profiling into the diagnostic process has significantly refined the classification and prognostication of non-glioblastoma astrocytomas. Mutations in the *isocitrate dehydrogenase* (*IDH*) genes (*IDH1*/*IDH2*) are a critical marker in these tumors, with IDH-mutant astrocytomas generally exhibiting a more favorable prognosis compared to their IDH-wildtype counterparts [21,22,23]. In addition, alterations such as *TP53* mutations and *ATRX* loss have been observed and are used to further stratify patients, potentially guiding therapeutic decision-making [24]. Recent reports also suggest that the presence of 1p/19q deletions, although more common in oligodendrogliomas, can occasionally be seen in mixed gliomas and may influence both the diagnostic categorization and the treatment approach [25].

The natural history of these tumors is indolent initially, yet there is a considerable risk for progression, particularly in Grade III lesions, which tend to have a more aggressive clinical course.

For low-grade astrocytomas, a combination of careful observation, radiotherapy, and, in some cases, chemotherapy (e.g., TMZ) may be employed, particularly if complete resection is not achievable [26]. Anaplastic astrocytomas, due to their higher risk of recurrence, often require a more aggressive treatment regimen that includes early adjuvant radiotherapy combined with chemotherapy.

Long-term follow-up is essential for these patients due to the risk of recurrence and progression. Advances in neuroimaging, molecular diagnostics, and surgical techniques continue to improve the management and outcomes of non-glioblastoma astrocytomas, although challenges remain in predicting individual tumor behavior and response to therapy.

#### 2.1.2. Glioblastoma

GBM is recognized as the most aggressive and lethal primary brain tumor in adults, classified as a Grade IV astrocytoma [4]. Characterized by rapid proliferation, diffuse infiltration, marked necrosis, and robust neo angiogenesis, GBM poses significant challenges in both surgical resection and long-term disease control [17]. In addition to these histopathological hallmarks, GBM exhibits a complex molecular landscape with numerous genetic alterations that influence tumor behavior, treatment response, and overall prognosis [27].

One of the most frequently encountered genetic alterations in GBM is the amplification of the *epidermal growth factor receptor* (*EGFR*) gene, which occurs in approximately 40% of cases [28,29]. This amplification often results in the expression of a mutant variant, EGFR^III^, which is constitutively active and contributes to enhanced tumor cell proliferation, survival, and resistance to conventional therapies. Moreover, mutations in *EGFR* can lead to aberrant downstream signaling through the PI3K/AKT and RAS/MAPK pathways, further promoting oncogenesis.

Another critical genetic alteration involves the tumor suppressor gene *PTEN*. Loss-of-function mutations or deletions in *PTEN* are observed in a significant subset of GBMs and are associated with unchecked cellular proliferation and survival due to deregulation of the PI3K/AKT signaling pathway [30,31]. In addition, mutations in *TP53* are also common, particularly in secondary GBMs that evolve from lower grade astrocytomas. *TP53* gene mutations impair the cell’s ability to undergo apoptosis in response to DNA damage, further contributing to tumor progression [32].

Mutations in the promoter region of the *telomerase reverse transcriptase* (*TERT*) gene represent another key molecular event in GBM. TERT promoter mutations are detected in a large proportion of GBM cases and lead to increased telomerase activity, which enables sustained replicative potential and cellular immortality [16].

Furthermore, although less common than in lower-grade gliomas, mutations in *isocitrate dehydrogenase* (*IDH*) are an important prognostic marker in GBM. *IDH* mutations, when present, are typically associated with secondary GBM and confer a relatively better prognosis compared to IDH-wildtype GBM. Metabolic reprogramming associated with mutant IDH, which includes the production of the oncometabolite 2-hydroxyglutarate, affects epigenetic regulation and cellular differentiation, thereby influencing tumor behavior and treatment response [21].

Other notable mutations include alterations in genes such as *NF1*, which can lead to dysregulation of RAS signaling, and aberrations in cell cycle regulators that further contribute to the aggressive phenotype of GBM [33]. The interplay between these genetic alterations not only underscores the intrinsic heterogeneity of GBM but also highlights potential targets for novel therapeutic approaches.

The current standard of care for GBM involves maximally safe surgical resection followed by a combination of radiotherapy and chemotherapy, typically with TMZ. However, despite these treatments, the median overall survival for patients remains dismal [17]. The presence of diverse molecular mutations within GBM tumors has spurred interest in targeted therapies. Additionally, immunotherapeutic approaches, including checkpoint inhibitors and vaccine-based strategies, are currently under investigation in clinical trials to overcome the tumor’s inherent resistance mechanisms [34].

Given the profound molecular heterogeneity and the adaptive resistance of GBM to current treatment strategies, there is a critical need for further studies. Future research must focus on elucidating the precise mechanisms by which these genetic alterations drive tumor progression and therapy resistance. In parallel, novel combination therapies that integrate targeted agents, immunotherapies, and conventional treatments should be explored to improve patient outcomes. Ultimately, a more personalized approach to GBM management, guided by comprehensive molecular profiling, will be essential to develop more effective, individualized treatment regimens for this formidable malignancy.

In conclusion, GBM’s dismal prognosis is a consequence of its aggressive histopathological features compounded by a complex array of molecular mutations, including *EGFR* amplification/mutation, *PTEN* loss, *TP53* mutation, *TERT* promoter mutations, and occasional *IDH* mutations. These genetic alterations not only drive tumorigenesis and treatment resistance but also serve as potential biomarkers and targets for future therapeutic interventions.

### 2.2. Meningioma

Meningiomas are the most common primary brain tumors, originating from the arachnoid cap cells of the meninges [35]. Meningothelial cells are specialized cells that form the arachnoid layer of the meninges, the protective coverings of the brain and spinal cord. These cells play a role in the production and regulation of cerebrospinal fluid, as well as in forming a barrier that helps protect the central nervous system [36]. When these cells undergo neoplastic transformation, they give rise to meningiomas [37]. This kind of tumor accounts for approximately one-third of all primary intracranial neoplasms and is most frequently diagnosed in middle-aged and elderly populations, with a higher prevalence in females. Although the majority of meningiomas are benign (Grade I) and exhibit slow growth, there exists a subset that demonstrates atypical (Grade II) or anaplastic (Grade III) behavior, which is associated with increased recurrence rates and poorer outcomes [38].

Meningiomas are often discovered incidentally during neuroimaging performed for unrelated reasons due to their typically indolent nature [39]. When symptomatic, patients may experience headaches, seizures, or focal neurological deficits, depending on the tumor’s size and location. Despite their benign nature, meningiomas can cause significant morbidity by exerting mass effect on adjacent brain structures, particularly when located at skull base regions or in proximity to critical neurovascular bundles [39].

Recent advancements in molecular diagnostics have shed light on the genetic alterations underlying meningioma pathogenesis. Mutations in the *neurofibromin 2* (*NF2*) gene are common, especially in sporadic meningiomas of the fibrous subtype, and are believed to play a pivotal role in tumor initiation [40,41]. Additionally, alterations in other genes such as *SMO*, *AKT1*, *TRAF7*, and *KLF4* have been identified, particularly in non-NF2 mutant meningiomas, indicating a heterogeneous molecular landscape that may influence both prognosis and treatment response [42].

The primary treatment for symptomatic meningiomas remains surgical resection, which is often curative for benign lesions [43]. However, complete resection may be challenging when tumors are located in eloquent brain areas or the skull base. In cases where the tumor is atypical or anaplastic, or when a subtotal resection is achieved, adjuvant radiotherapy is frequently employed to reduce the risk of recurrence [43].

Immunotherapeutic approaches are also emerging as potential adjuncts in treating high-grade or recurrent meningiomas, although these strategies are still in the early stages of clinical evaluation [44].

The integration of molecular profiling into routine clinical practice holds promise for refining the classification and management of meningiomas [14]. A better understanding of the molecular drivers of tumor progression and recurrence, such as the roles of NF2, SMO, AKT1, TRAF7, and KLF4 mutations, will facilitate the development of targeted therapies, potentially improving outcomes for patients with atypical and anaplastic meningiomas [45]. Moreover, ongoing research is needed to elucidate the contributions of the TME, including immune cell infiltration and angiogenesis, to meningioma pathobiology, which may reveal novel therapeutic targets and strategies to overcome resistance to current treatments [14]. These research efforts are critical for developing more effective, personalized treatment approaches that not only improve long-term outcomes but also enhance the quality of life for patients afflicted with this diverse group of neoplasms.

Ongoing research is essential to address the intrinsic heterogeneity of these tumors and to translate molecular discoveries into effective treatments.

### 2.3. Pediatric Brain Tumors

Pediatric brain tumors represent a distinct category of CNS neoplasms, differing significantly from adult brain tumors in terms of histology, molecular profile, clinical behavior, and treatment response [46]. The most common types in children include medulloblastomas, ependymomas, atypical teratoid/rhabdoid tumors (AT/RT), pilocytic astrocytomas, and diffuse midline gliomas [47]. These tumors often arise in different anatomical locations compared to adults, with a predilection for the posterior fossa and brainstem, which can complicate surgical approaches. Molecular characterization has revealed unique alterations in pediatric tumors, such as mutations in the *SMARCB1* gene in AT/RT, and *H3K27M* mutations in diffuse midline gliomas [48,49].

The clinical course of pediatric brain tumors also differs from adults: low-grade tumors, such as pilocytic astrocytomas, may have excellent long-term survival with complete resection, whereas high-grade tumors, including diffuse intrinsic pontine gliomas, carry a very poor prognosis despite aggressive therapy [50]. Treatment strategies often combine surgery, radiotherapy, and chemotherapy, tailored to tumor type, molecular features, and the child’s age, with special attention to minimizing long-term neurocognitive and developmental side effects. Emerging therapies, including targeted agents, immunotherapies, and, in selected high-grade cases, tumor treating fields, are under investigation to improve outcomes while reducing toxicity [51].

Overall, pediatric brain tumors require a multidisciplinary approach integrating neuroimaging, molecular diagnostics, and individualized treatment planning, highlighting the importance of understanding tumor biology.

## 3. Gut–Brain Axis: A Complex Communication Network

The gut–brain axis is an intricate and multifaceted communication network that links the gut and the brain [52]. This bidirectional system enables constant interaction between the two organs, influencing physiological processes, behavior, and mental health [53]. The axis involves multiple pathways, including the CNS, the enteric nervous system (ENS), hormones, immune responses, and even the microbiota residing in the gut. It is often described as a “second brain” due to its ability to function autonomously and communicate directly with the CNS [52]. One of the main mechanisms through which the gut and brain interact is the vagus nerve, which acts as the primary communication route between the gut and the brain [53]. The microbiota within the gut also plays a crucial role in modulating the signals transmitted through this pathway, sending chemical messages that can affect brain function and behavior. Hormones like cortisol, serotonin, and dopamine, which are produced or regulated in the gut, also act as signaling molecules that influence both mood and cognitive functions [1]. Additionally, the gut microbiota interacts with the immune cells in the gut lining, impacting the immune response and potentially influencing neuroinflammation [2,54]. This complex interaction between the gut microbiota, immune system, and brain highlights the importance of maintaining a balanced gut microbiota for optimal brain health. The gut–brain axis is a field of growing interest in research, as scientists continue to uncover how disturbances in this network may contribute to various neurological disorders.

### Effects of Gut Microbiota on Brain Health

Recent research has explained the profound influence that gut microbiota exerts on brain health [2,53,54,55,56]. The human gut is home to trillions of microorganisms, including bacteria, fungi, and viruses, that form a diverse and dynamic microbiome. These microbes are involved in numerous physiological functions, ranging from nutrient metabolism to immune modulation [57]. The microbiota plays a crucial role in the synthesis and regulation of key neurotransmitters that are essential for brain function [58,59]. For instance, the gut microbiota influences the production and release of serotonin, a neurotransmitter that regulates mood, sleep, and appetite, which can impact mood and emotional responses [60,61]. Dysregulation of the microbiota–brain axis has been implicated in the development of several neurological and psychiatric disorders, including depression, anxiety, migraine, and autism spectrum disorders (ASD) [61,62].

**Figure 1:**
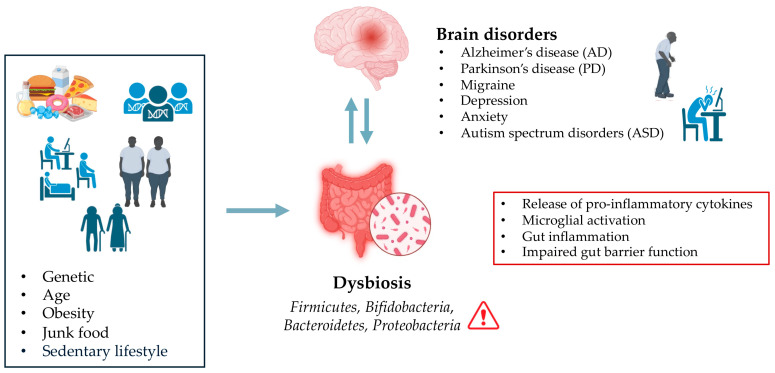
Effect of Dysbiosis on Brain Disorders.

Moreover, studies have shown that an imbalance in gut microbial communities, known as dysbiosis, is associated with the development of neurogenerative diseases such as Alzheimer’s disease (AD) and Parkinson’s disease (PD) [3,63,64,65] (Figure 1).

Inflammatory responses triggered by an imbalanced gut microbiota are believed to contribute to brain inflammation, which can exacerbate neurodegenerative processes. The interaction between gut-derived inflammatory mediators and the brain’s immune system can lead to chronic low-grade inflammation, which is often observed in conditions like AD and PD. In the context of AD, a systematic review conducted by Manfredi et al. [66] showed a decreased abundance of *Firmicutes* and *Bifidobacteria*, and an increase in Bacteroidetes and *Proteobacteria* in both human and mouse AD models. These alterations in gut microbiota were found to exacerbate AD pathology by increasing also BBB permeability [66]. Another report on Egyptian patients with PD (*n* = 30) showed significant alterations in gut microbiota composition compared to healthy controls (*n* = 35) [67]. PD patients displayed a reduction in *Firmicutes* and *Bifidobacteria*, and an increase in *Bacteroides*. The *Firmicutes*/*Bacteroidetes* ratio was markedly lower in PD patients (0.62) than the healthy control (1.32) [67]. Importantly, microbial profiles correlated with clinical PD subtypes: tremors predominant had lower *Firmicutes* and *Firmicutes*/*Bacteroidetes* ratio, while both tremors and postural instability and gait disability (PIGD) phenotypes had lower *Bifidobacteria* [67].

As mentioned earlier, the gut microbiota can influence the integrity of the BBB, which controls the entry of substances into the brain [7]. The BBB’s integrity is maintained by tight junction (TJ) proteins, such as claudin-5, occludin, and ZO-1 [7], which form the structural basis of its selective permeability [7]. Disruptions in the microbiota can compromise BBB function, potentially allowing harmful substances, such as pathogens or toxins, to cross into the brain and damage neural tissue, thereby promoting a neuroinflammatory state [7].

The gut microbiota also has a direct role in regulating the autonomic nervous system (ANS), which in turn controls several functions such as heart rate, digestion, and respiratory rate. By influencing the ANS, the gut microbiome can affect not just brain function but also the physical aspects of brain health, such as blood flow and cellular repair mechanisms [68]. Additionally, microbiota-derived metabolites, such as SCFAs, play a key role in maintaining brain health by supporting neuronal survival and modulating neuroinflammation [62,69].

Given the significant effects of the gut microbiota on brain health, researchers are exploring probiotic and prebiotic therapies to promote a healthy gut microbiota as potential treatments for various neurological and psychological conditions. Probiotics, which are live beneficial bacteria, and prebiotics, which are compounds that promote the growth of beneficial gut bacteria, are being tested for their ability to restore balance to the gut microbiota and alleviate symptoms of mental health disorders [62,69,70]. Emerging findings suggest that modulating the microbiota through diet, supplements, and lifestyle changes could be a promising strategy to prevent or treat mental health disorders and neurodegenerative diseases [62,71,72,73]. For instance, diets rich in fiber, fermented foods, and polyphenols have been shown to support the growth of beneficial gut bacteria, while reducing the abundance of harmful bacteria linked to conditions like depression and anxiety [73].

In addition to diet, recent studies are investigating the use of FMT as a potential treatment for restoring gut health in certain neurological conditions [9,65,74]. By transferring healthy microbiota from a donor to a recipient, FMT aims to re-establish microbial balance and alleviate symptoms associated with neurological dysfunction.

While microbiota-based therapies hold promise, their effectiveness ultimately depends on the molecular pathways by which gut microbes shape brain function, many of which converge on key immune and inflammatory signaling cascades and are promising for the development of future treatments.

For instance, emerging evidence supports a central role for the NLRP3 inflammasome as a molecular hub in the gut-microbiota–brain axis, mediating the bidirectional crosstalk between dysbiosis and neuroinflammation [75]. Gut-derived signals, such as LPS, extracellular ATP, reactive oxygen species (ROS), β-amyloid, and α-synuclein aggregates, can trigger NLRP3 activation via pattern recognition receptor (PRR)-mediated pathways in both intestinal and central innate immune cells, including macrophages and microglia [76].

Once activated, the NLRP3 inflammasome assembles a complex with ASC and pro-caspase-1, leading to caspase-1 cleavage and maturation of IL-1β and IL-18, culminating in neuroinflammation and pyroptotic cell death, which exacerbate BBB disruption and neuronal injury [77].

This activation loop is further compounded by gut dysbiosis: in animal models, NLRP3 knockout or caspase-1 deficiency alters microbiota composition, behavioral outcomes, and gut motility, underscoring a mutual regulatory feedback loop between microbiota and the inflammasome [78].

In pathological contexts such as AD, PD, major depressive disorder (MDD), and intracerebral hemorrhage (ICH), microbiota-driven activation of NLRP3 promotes peripheral and central inflammation, compromises gut barrier integrity, and leads to white matter injury and neurodegeneration [79].

Notably, in models of ICH, dysbiosis-induced NLRP3 activation mediates secondary injury by disrupting the BBB, inducing neuroinflammation, and hindering nerve regeneration [80]; pharmacological inhibition of NLRP3 (e.g., with MCC950) has shown protective effects on white matter integrity and cognitive function [6].

Collectively, current evidence suggests that gut dysbiosis may drive NLRP3 over-activation, fostering the release of pro-inflammatory cytokines, which in turn may further alter gut microbial composition, thus perpetuating a vicious cycle of dysbiosis and neuroinflammation [81].

As the understanding of the gut–brain connection deepens, researchers continue to explore novel ways to leverage the gut microbiome in the treatment and prevention of a wide range of neurological and psychological disorders. The emerging field of microbiome-based medicine may offer new hope for improving brain health.

## 4. The Role of Gut Microbiota in Brain Tumor Development, Progression, and Treatment

Recent research into the gut–brain axis has opened up new avenues for understanding how the gut microbiota, the complex community of microorganisms residing in the GI tract, can influence brain health [62,82]. Traditionally, the gut microbiota has been linked to mental health conditions, including mood disorders, and neurodegenerative diseases [83]; however, emerging evidence suggests it also plays a crucial role in the development, progression, and treatment of brain tumors [56,84] (Table 2).

**Figure 2:**
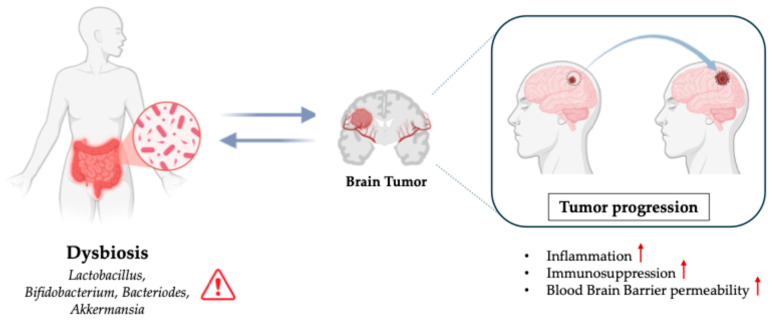
Role of Dysbiosis in Brain Tumors.

Brain tumors, particularly gliomas, are one of the most devastating types of cancer, with a high mortality rate and limited treatment options [11]. Investigating how the gut microbiota interacts with CNS and brain tumor environments could provide groundbreaking insights into new therapeutic strategies (Figure 2).

### 4.1. Gut Microbiota and Brain Tumor Development

The interplay between gut microbiota, inflammatory state and brain tumor development is complex and multifactorial [2,82]. Recent studies suggest that microbial communities in the gut influence the onset of cancer, including brain tumors, through various mechanisms [2,8,85,88]. Tumor cells within the brain can alter the local microenvironment, and emerging evidence suggests that microbial imbalances may influence tumor growth indirectly by affecting systemic inflammation, immune responses, and metabolic pathways [89]. One of the key mechanisms through which the gut microbiota might influence brain tumor development is via the modulation of the immune system. An imbalance in the gut microbiota can lead to immune system dysfunction and chronic inflammation, which is a known contributor to the development of many cancers, including gliomas [2,90]. Studies have shown that a dysregulated microbiota promotes an inflammatory state that may support the initiation of tumorigenesis [82,91]. For example, an overgrowth of pro-inflammatory bacteria or the depletion of beneficial bacteria might result in an immune response that fosters the growth of abnormal cells in the brain [92]. Gut-derived metabolites such as SCFAs, which are produced by beneficial gut bacteria, may have tumor-suppressive properties [93]. SCFAs play a key role in modulating immune responses and neuroinflammation, even influencing tumor suppressor gene expression [72,93,94,95]. Dysbiosis in the gut microbiota may disrupt the production of these metabolites, potentially creating a microenvironment conducive to the initiation of brain tumors [96].

### 4.2. Gut Microbiota and Brain Tumor Progression

Once a brain tumor has developed, the role of the gut microbiota in tumor progression becomes even more pronounced. The gut microbiota can impact tumor growth, metastasis, and the immune landscape of the brain TME through several mechanisms [84,97]. The immune system plays a dual role in cancer as it can suppress or promote tumor growth by allowing for immune evasion or aiding in tumor immune tolerance [89,98]. In gliomas, for example, immune checkpoints such as PD-1/PD-L1 and CTLA-4 are often upregulated, suggesting a worse outcome [99,100]. It is believed that the gut microbiota can influence the expression of these checkpoints by modulating systemic inflammation and immune cell activity [101]. Certain gut bacteria, including *Bifidobacterium* and *Akkermansia*, have been found to enhance the efficacy of immunotherapies by stimulating anti-tumor immune responses [5]. Moreover, microbiota can impact the TME by the production of metabolites like SCFAs that regulate cellular metabolism and inflammation [56,86,93]. For example, butyrate, a major SCFA, has been shown to inhibit tumor proliferation and reduce invasiveness through modulation of cell cycle progression and even affecting extracellular matrix substrates [102,103].

### 4.3. Gut Microbiota and Brain Tumor Treatment

The influence of the gut microbiota on brain tumor treatment is an emerging area of research with significant implications for therapeutic strategies. Traditional treatments for brain tumors, such as surgery, radiation, and chemotherapy, have limited success due to the BBB and the complexity of the TME [104].

The gut microbiota contributes to human health through multiple mechanisms, such as the digestion of complex carbohydrates, modulation of nutrient absorption, secretion of microbial metabolites (e.g., SCFAs, lipopolysaccharide (LPS)), and the production of vitamins and neurotransmitters [105]. Increasing evidence indicates that these processes extend beyond the intestine, influencing CNS function and brain physiology. In particular, the gut microbiota plays a critical role in maintaining immune homeostasis in the brain by modulating the activity of microglia, T cells, dendritic cells (DCs), macrophages, and other immune cells [106].

In the oncological context, alterations in gut microbiota composition have been shown to influence tumor progression and therapeutic outcomes [2,10,87,107,108].

Notably, previous studies have suggested an association between commensal gut microbe’s alterations and brain tumors, including GBM [96,109]. For instance, Wang et al. [109] reported that higher abundances of *Ruminococcaceae* were associated with a reduced risk of developing GBM. Members of this family synthesize the metabolite isoamylamine (IAA), which promotes microglia activation by enhancing the recruitment of p53, a key transcription factor involved in cell cycle regulation, DNA repair, and apoptosis, to the S100A8 promoter region [110].

Probiotics, particularly those containing *Lactobacillus* and *Bifidobacterium* strains, are being investigated for their potential to enhance anti-tumor response [70,111,112].

A study by Wang et al. [88] demonstrated that supplementation with *Bifidobacterium lactis* and *Lactobacillus plantarum* reduced tumor volume, prolonged survival, and improved intestinal barrier integrity by modulating TJs expression in an orthotopic glioma mouse model.

Among the most promising avenues of investigation is the role of the gut microbiota in enhancing the efficacy of immunotherapies for brain tumors [87,107].

By modulating innate and adaptive immune responses through metabolite production and other regulatory pathways, the microbiota has been shown to influence the effectiveness of immune checkpoint inhibitors (ICIs) and related therapies [2].

For example, Dees and colleagues [113] demonstrated in a murine glioma model with a humanized microbiome that microbial composition influences the success of anti-PD-1 therapy.

In detail, fecal material from five healthy human donors was transplanted into gnotobiotic mice [113]. Once the transplanted microbiomes stabilized, the mice were bred to generate five independent humanized mouse lines (HuM1–HuM5). All HuM lines were susceptible to GBM transplantation and exhibited comparable median survival times ranging from 19 to 26 days. The response to anti-PD-1 treatment varied among the lines: HuM1, HuM4, and HuM5 were non-responders, whereas HuM2 and HuM3 were responsive, showing a significantly prolonged survival compared with isotype-treated controls. Bray–Curtis cluster analysis revealed that the gut microbial communities of responder lines HuM2 and HuM3 were closely related [113]. Further taxonomic comparison identified *Bacteroides cellulosilyticus* as a common species enriched in HuM2 and HuM3, suggesting a potential role in mediating sensitivity to immune checkpoint inhibition [113].

Commensal bacteria shape both innate and adaptive immunity, with microbial metabolites such as SCFAs, which in turn modulate inflammatory pathways and immune cell recruitment [103].

SCFAs, including acetate, propionate, and butyrate, are produced by the fermentation of dietary fibers. SCFAs are known to exert beneficial effects in the context of neuroinflammatory diseases [62,69,114,115,116,117] as well as in brain tumors, reducing GBM cells proliferation by modulating several signaling pathways [72,106,118].

Among SCFAs, butyrate accounts for approximately 20% of the total SCFAs in the gut and plays key roles in energy supply to intestinal epithelial cells, as well as in cellular regulation, proliferation, and differentiation [106]. Most butyrate-producing bacteria belong to the phyla *Firmicutes*, *Actinobacteria*, *Bacteroidetes*, *Fusobacteria*, and *Proteobacteria*. Butyrate is known to influence innate immunity by promoting monocyte differentiation into macrophages, which can polarize into pro-inflammatory M1 or immunosuppressive M2 phenotypes, contributing to tumor progression [119]. In this context, Zhou et al. [56] demonstrated by using an orthotopic GBM model that gut dysbiosis caused by antibiotic treatment accelerated glioma growth by increasing the percentage of M2-like macrophage populations and reducing the levels of gut microbial metabolites SCFAs in the TME. In detail, 21 days after tumor implantation, the authors performed 16Sr RNA sequencing on fecal samples from the cecum of GL261-bearing mice, showing that the numbers of the most abundant microbiota, like *Firmicutes* and *Bacteroidetes* were decreased or vanished, but the numbers of *Proteobacteria* were increased in the gut microbiota of ABX-treated tumor-bearing mice [56]. However, oral supplementation of SCFAs reversed these effects by shifting macrophages toward an M1-like phenotype, resulting in an enhanced M1/M2 ratio and improving glioma outcomes [56] (Table 3).

Another crucial mediator involved in the gut–brain axis is LPS, a pro-inflammatory endotoxin derived from Gram-negative bacteria, that can trigger systemic inflammation by activating Toll-like receptor 4 (TLR4) [120,121,122]. Elevated LPS levels have been associated with increased inflammation in cancer and brain diseases [121,123,124,125]. Because TLRs can modulate both innate and adaptive immunity, TLR ligands are a promising approach for brain tumor immunotherapy. Accordingly, LPS, as a well-known TLR4 ligand, has been reported to alter the immuno-phenotype of glioma and glioma stem-like cells, and induce anti-tumor effects [126,127].

In recent years, cancer vaccines are an emerging and rapidly evolving area in immuno-oncology, aiming to stimulate the immune system to recognize and eliminate tumor cells by presenting tumor-associated antigens (TAAs) [128]. In GBM, a notoriously immunosuppressive tumor, several vaccine strategies have shown promise in enhancing immune responses. One innovative approach involves the use of an amphiphile-ligand conjugated with a tumor peptide vaccine. In preclinical models of EGFRvIII-positive GBM, this system traffics from the bloodstream to lymph nodes, where it integrates into the membranes of resident antigen-presenting cells (APCs) [128,129]. This process efficiently primes both CD4^+^ and CD8^+^ T cells and promotes antigen spreading, thereby enhancing the efficacy of adoptive cell therapies such as CAR-T cells. Another well-established strategy is the use of DC vaccines, which are generated ex vivo by differentiating monocytes or hematopoietic progenitor cells into mature DCs and loading them with tumor antigens either synthetic peptides or autologous tumor lysates [130]. These antigen-loaded DCs activate CD4^+^ T-helper cells and, through cross-priming, cytotoxic CD8^+^ T cells. Synthetic peptides are particularly efficient at enhancing cytotoxic T lymphocyte (CTL) responses due to their rapid processing and presentation by DCs. Accordingly, clinical trials have demonstrated encouraging outcomes; notably, the phase III DCVax-L trial reported significant improvements in overall survival in both newly diagnosed and recurrent GBM patients [128,131].

Another strategy includes PVSRIPO, a genetically modified, nonpathogenic poliovirus engineered to target CD155, a receptor commonly overexpressed in GBM cells. Upon direct intratumoral administration, PVSRIPO selectively infects and lyses tumor cells, while simultaneously activating strong local and systemic immune responses. This virotherapy not only exerts direct cytotoxicity but also acts as an in situ vaccine by releasing tumor antigens and promoting immune activation [132]. These vaccine-based therapies—whether bacterial, viral, or cell-based—offer a promising complement to conventional GBM treatments, including surgery, radiation, and chemotherapy.

The gut microbiota is increasingly recognized as a critical modulator of therapeutic outcomes. Microbiome-based interventions, such as targeted probiotics, may further enhance the efficacy of cancer vaccines by shaping systemic and intratumoral immunity. As research continues to uncover the complex interplay between the microbiota, the immune system, and tumor biology, microbiota-informed vaccine strategies may open new therapeutic avenues for GBM.

## 5. Studies on the Influence of Microbiota in Brain Tumors

The burgeoning field of microbiota research has led to an increasing focus on the gut–brain axis, particularly its role in influencing brain tumor development, progression, and treatment. A growing body of studies has started to unravel the intricate relationship between gut microbiota and brain tumors, particularly gliomas, which are among the most aggressive types of brain cancers [2,8]. These studies are illuminating how the gut microbiota can shape the brain TME, modulate immune responses, and affect the efficacy of therapies [2,8]. Understanding these interactions could potentially lead to the development of new treatment strategies that involve microbiota manipulation to enhance treatment outcomes in brain cancer patients. For instance, Fan et al. [8] explored the relationship between gut microbiome and glioma progression in mice, demonstrating that gut microbiota dysbiosis downregulates Foxp3 expression in the brain, promoting glioma growth by modulating the Foxp3 signaling pathway. In another study, D’Alessandro et al. [2] investigated the effect of gut microbiota alteration in a syngeneic mouse model of glioma, treating mice with two antibiotics (ABX) and evaluating their effects on tumor growth, microbe composition, natural killer (NK) cells, and microglia phenotype. They demonstrated, in this study, that ABX treatment altered the intestinal microbiota, reduced cytotoxic NK cell subsets, and altered the expression of inflammatory and homeostatic proteins in microglia, contributing to glioma growth. These results reveal that chronic ABX administration alters microbiota composition and contributes to modulating the brain’s immune state, paving the way to glioma growth.

As previously explained, the gut microbiome is essential in neurogenesis processes. Alterations in microbial constituents could promote inflammation and immunosuppression. Recently, in immune-oncology, specific microbial taxa have been described to enhance the effects of therapeutic treatment. In this context, Patrizz et al. [85] evaluated the effects of glioma development and TMZ on fecal microbiome in mice and humans. This study found that glioma development alters gut microbiota composition in both mice and humans, increasing the *Firmicutes*/*Bacteroidetes* (F/B) ratio and *Akkermansia* levels. TMZ treatment affected microbiota in non-tumor-bearing mice but did not replicate glioma-induced changes. The findings suggest a link between glioma and gut dysbiosis, warranting further research.

While preclinical studies have provided compelling evidence of the gut microbiota’s influence on brain tumor biology, translating these findings to human patients is an essential next step in determining the clinical relevance of microbiome modulation in brain cancer treatment.

A growing body of evidence suggests that the microbiota’s influence extends beyond just the immune system, potentially affecting cancer cell metabolism, inflammation, and the BBB that can ultimately alter treatment outcomes.

While these early clinical findings are promising, more large-scale, randomized clinical trials are needed to definitively establish the role of the microbiota in brain tumor treatment.

## 6. Conclusions and Future Perspectives

In conclusion, the gut microbiota plays a significant role in the development, progression, and treatment of brain tumors. By influencing immune responses, tumor metabolism, and the response to therapies, the microbiota can either promote or inhibit tumor growth. As research into the gut–brain axis continues to unfold, it is becoming increasingly clear that microbial communities in the gut are integral to brain tumor biology. One of the most promising future directions could involve the potential use of the gut microbiota as a diagnostic tool for brain tumors. For instance, changes in the diversity and composition of gut bacteria may precede the onset of brain tumors, and specific microbial profiles could be linked to the presence of tumors or the stage of tumor progression. Advances in metagenomics and microbiome sequencing techniques could enable clinicians to identify these microbial markers and offer personalized, early detection methods for high-risk individuals. Moreover, by analyzing the patient’s unique gut microbiome composition, it may be possible to customize treatment plans that consider the microbial influence on tumor response.

## Figures and Tables

**Table 1:** Classification of Gliomas According to WHO.

WHO Grade	Glioma Type	Key Characteristics	Aggressiveness	Key Diagnostic Genes
Grade I	Pilocytic Astrocytoma	Solid, well-circumscribed, non-infiltrative	Low (benign)	*KIAA1549-BRAF*, *BRAF*, *NF1*
	Subependymal Giant Cell Astrocytoma (SEGA)	Associated with tuberous sclerosis, slow growing	Low (benign)	*TSC1*, *TSC2*
Grade II	Diffuse Astrocytoma	Infiltrative, slow growing, may progress to higher grades	Intermediate	*MYB*, *MYBL1*
	Oligodendroglioma	Infiltrative	Intermediate	*IDH1*, *IDH2*, 1p/19q, *TERT* promoter, *CIC*, *FUBP1*, *NOTCH1*
Grade III	Anaplastic Astrocytoma	Infiltrative, more aggressive than Grade II	High (malignant)	*IDH1*/*2*, *ATRX*, *TP53*
	Anaplastic Oligodendroglioma	Infiltrative, with genetic features typical of oligodendrogliomas	High (malignant)	*IDH1*/*IDH2* mutations, *TERT*, *CIC*, *FUBP1*
Grade IV	GBM	Highly infiltrative, necrosis, and microvascular proliferation	Very high (malignant)	IDH-wildtype, *TERT* promoter, chromosomes 7/10, *EGFR*

GBM, Glioblastoma.

**Table 2:** Role of Gut Microbiota in Brain Tumor Development, Progression, and Treatment.

Phase	Role of Gut Microbiota	References
Tumor Development	Microbiota dysbiosis contributes to neuroinflammation and tumor initiation	[8,85]
Tumor Progression	Gut microbiota influences TME, immune response, and metabolic changes that promote glioma growth	[2,86]
Therapeutic Response	Microbiota affects the efficacy of chemotherapy, radiotherapy, and immunotherapy	[10,84,87]

TME, tumor microenvironment.

**Table 3:** Role of Gut Microbiota in Brain Tumor Treatment.

Gut Microbiota Component/Intervention	Mechanism/Pathway	Effect on Brain Tumors	References
*Ruminococcaceae*	Synthesizes isoamylamine (IAA) and activates microglia via p53 recruitment	Reduced risk of GBM development	[109]
*Bifidobacterium lactis* and *Lactobacillus plantarum*	Modulate PI3K/AKT pathway and TJ expression	Reduced tumor growth and increased survival in an orthotopic glioma mouse model	[88]
*Bacteroides cellulosilyticus*	Modulates innate and adaptive immunity via metabolites and regulatory pathways	Potential mediator of sensitivity to anti-PD-1 therapies	[113]
SCFA supplementation	Restores macrophage M1/M2 balance	Improved glioma outcomes and reversed effects of gut dysbiosis	[56]

GBM, Glioblastoma.

## Data Availability

Not applicable.
